# Alterations in electroencephalographic functional connectivity in individuals with major depressive disorder: a resting-state electroencephalogram study

**DOI:** 10.3389/fnins.2024.1412591

**Published:** 2024-07-10

**Authors:** Yingtan Wang, Yu Chen, Yi Cui, Tong Zhao, Bin Wang, Yunxi Zheng, Yanping Ren, Sha Sha, Yuxiang Yan, Xixi Zhao, Ling Zhang, Gang Wang

**Affiliations:** ^1^National Clinical Research Center for Mental Disorders and National Center for Mental Disorders, Beijing Key Laboratory of Mental Disorders, Beijing Anding Hospital, Capital Medical University, Beijing, China; ^2^Advanced Innovation Center for Human Brain Protection, Capital Medical University, Beijing, China; ^3^Gnosis Healthineer Co. Ltd, Beijing, China

**Keywords:** major depressive disorder, electroencephalography, functional connectivity, phase locking value, neural oscillation

## Abstract

**Background:**

Major depressive disorder (MDD) is the leading cause of disability among all mental illnesses with increasing prevalence. The diagnosis of MDD is susceptible to interference by several factors, which has led to a trend of exploring objective biomarkers. Electroencephalography (EEG) is a non-invasive procedure that is being gradually applied to detect and diagnose MDD through some features such as functional connectivity (FC).

**Methods:**

In this research, we analyzed the resting-state EEG of patients with MDD and healthy controls (HCs) in both eyes-open (EO) and eyes-closed (EC) conditions. The phase locking value (PLV) method was utilized to explore the connection and synchronization of neuronal activities spatiotemporally between different brain regions. We compared the PLV between participants with MDD and HCs in five frequency bands (theta, 4–8 Hz; alpha, 8–12 Hz; beta1, 12–16 Hz; beta2, 16–24 Hz; and beta3, 24–40 Hz) and further analyzed the correlation between the PLV of connections with significant differences and the severity of depression (via the scores of 17-item Hamilton Depression Rating Scale, HDRS-17).

**Results:**

During the EO period, lower PLVs were found in the right temporal-left midline occipital cortex (RT-LMOC; theta, alpha, beta1, and beta2) and posterior parietal-right temporal cortex (PP-RT; beta1 and beta2) in the MDD group compared with the HC group, while PLVs were higher in the MDD group in LT-LMOC (beta2). During the EC period, for the MDD group, lower theta and beta (beta1, beta2, and beta3) PLVs were found in PP-RT, as well as lower theta, alpha, and beta (beta1, beta2, and beta3) PLVs in RT-LMOC. Additionally, in the left midline frontal cortex-right temporal cortex (LMFC-RT) and posterior parietal cortex-right temporal cortex (PP-RMOC), higher PLVs were observed in beta2. There were no significant correlations between PLVs and HDRS-17 scores when connections with significantly different PLVs (all *p* > 0.05) were checked.

**Conclusion:**

Our study confirmed the presence of differences in FC between patients with MDD and healthy individuals. Lower PLVs in the connection of the right temporal-left occipital cortex were mostly observed, whereas an increase in PLVs was observed in patients with MDD in the connections of the left temporal with occipital lobe (EO), the circuits of the frontal-temporal lobe, and the parietal-occipital lobe. The trends in FC involved in this study were not correlated with the level of depression.

**Limitations:**

The study was limited due to the lack of further analysis of confounding factors and follow-up data. Future studies with large-sampled and long-term designs are needed to further explore the distinguishable features of EEG FC in individuals with MDD.

## 1 Introduction

Major depressive disorder (MDD) is characterized by persistent feelings of sadness, hopelessness, and a loss of interest or pleasure in daily activities; some patients might have recurrent thoughts of death (Marx et al., 2023). According to recent epidemiological data, cases of MDD are estimated to increase to 53.2 million due to the COVID-19 pandemic. The prevalence of people with MDD has increased to 3,152.9 cases per 100,000 population, with disability-adjusted life years (DALYs) reaching 49.4 million, suggesting that MDD, among all mental illnesses, is the leading cause of disability (COVID—Mental Disorders Collaborators, 2021; GBD Mental Disorders Collaborators, 2022). The diagnosis of MDD is mainly based on clinical assessments (including mental state scales or tools), history taking from patients, face-to-face evaluation by psychiatrists, and references to diagnostic criteria or guidelines (American Psychiatric Association, 2022; World Health Organization, 2024). However, the constant presence of subjectivity may influence the accuracy of the diagnosis (Del-Ben et al., 2005). This situation has prompted researchers to explore objective indicators of MDD diagnosis. By doing so, it is possible to avoid the interference of subjectivity in the diagnostic process. Over the years, massive depression-related biomarkers with potential applications in the diagnosis have been discovered (Etkin et al., 2015; Takahashi et al., 2017; Chang et al., 2018; Humphreys et al., 2019; Jones and Nemeroff, 2021), although the range of their applications is relatively narrow and there are still many challenges in their usability, maneuverability, and stability.

Although the pathogenesis remains to be clarified, MDD is considered to be significantly associated with human brains, and more attention has been paid to this area. It has been verified that the emotions of humans can be formed on a scale of hundreds of milliseconds (Hari and Parkkonen, 2015). Electroencephalography (EEG), as a non-invasive, low-cost, and convenient procedure, is quite accessible for detecting valuable brainwave features with high temporal resolution at the millisecond level; thus, EEG has been gradually applied to explore the possibilities of detecting and diagnosing mental illnesses such as MDD (Feldmann et al., 2018). Researchers have developed and applied a variety of analysis methods to identify MDD patients. Mumtaz et al. (2017) differentiated MDD and normal controls using clinical features extracted from EEG. Liao et al. (2017) proposed a method based on EEG signals and a spectral-spatial feature extractor named kernel eigen-filter-bank common spatial pattern, and they achieved an average classification accuracy of 81.23%. Acharya et al. (2015) created a depression diagnosis index by using non-linear features and reported an average accuracy of 98%. Previous findings suggest that some indicators of EEG might be promising biomarkers, while for our current research, functional connectivity (FC) could be the one that shows significant differences.

FC is defined as the temporal correlation among the activities of different neural assemblies; such a correlation originates from statistically significant dependence between distant brain regions. FC mainly reflects the synchronization of two different electrode pairs (Fingelkurts et al., 2005; Sakkalis, 2011). It has been found that FC could be influenced by white matter myelinated cortico-cortical axons as it originates from post-synaptic potentials (Hall et al., 2014; Nunez et al., 2015). Myelin, however, is in charge of the axon, controlling its speed and the synchrony of impulse traffic between different cortical regions, which is fairly important for optimal mental performance (Nunez et al., 2015). Considering that the distances of brain signals are various, it is quite essential to ensure that the signals would reach their target simultaneously, and such a model of connections might explain the wide range of EEG frequency bands (Nunez et al., 2015). There are various measures of FC, including coherence (Han et al., 2021), correlation coefficient, amplitude envelope correlation, phase lag index, weighted phase lag index, synchronization likelihood, and phase locking value (PLV). EEG has been broadly utilized for the analysis of FC in individuals with MDD.

Previous studies have extensively investigated FC in different frequency bands of EEG between individuals with MDD and healthy controls (HCs). For the delta band, a majority of studies did not find any difference (Olbrich et al., 2014; Knyazev et al., 2018; Whitton et al., 2018). Knyazev et al. (2018) reported no difference in FC between individuals with MDD and HCs. Only Leuchter et al.'s study revealed that the FC of individuals with MDD was relatively higher in limited connections (Leuchter et al., 2012). Low connectivity was still observed in individuals with MDD (McVoy et al., 2019; Hasanzadeh et al., 2020). For the gamma band, none of the available findings showed any significant difference between individuals with MDD and HCs (Park et al., 2007; Knyazev et al., 2018; Whitton et al., 2018). For alpha, high-quality studies conducted by Fingelkurts et al. revealed higher FC in individuals with MDD (Fingelkurts et al., 2007; Fingelkurts and Fingelkurts, 2017), whereas FC was observed to be lower in the findings of Iseger et al. (2017), Knyazev et al. (2018), and Whitton et al. (2018). For the theta band, the results from Fingelkurts's study still showed higher FC in patients with MDD than in HCs (Fingelkurts et al., 2007), but the results of some other studies, such as the findings from Sun et al., presented a completely opposite dynamic (Sun et al., 2019). For beta, most previous studies indicated significant differences between individuals with MDD and HCs, such as the findings from Hasanzadeh et al. (2020), Knyazev et al. (2018), and Leuchter et al. (2012), which revealed relatively higher FC for individuals with MDD. However, some studies reported that patients with depression show lower FC than HCs (Knott et al., 2001; McVoy et al., 2019). Considering the existence of heterogeneity in the method of processing and analysis, as well as the sample sizes that are small or have large differences between groups, more convincing research from the perspective of neurophysiologic is needed, through which we could further discover the essence of pathogenic mechanism in MDD.

Our study focused on the connectivity analysis of resting-state EEG and aimed to observe whether and how the connectivity features of patients with MDD differ from HCs during depressive episodes. We chose to measure PLV, which has been widely used to quantify the correlation between electrodes, to bring deeper insights into the FC and synchronization between different brain regions. As PLV is an indicator containing phase information, it is believed that the measure can reflect FC and synchronization in neuronal activities from the perspective of both time and space. Such spatiotemporal changes can be observed independent of amplitude characteristics. The shift is highly correlated with emotional activity (Cui et al., 2023). In this study, we compared the PLV of EEG in patients with MDD and HCs at different frequency bands and further explored its relationship with the severity of depressive symptoms.

## 2 Method

### 2.1 Subjects and participants

The research was conducted among patients admitted at Beijing Anding Hospital, and healthy subjects were openly recruited through advertisement and social media. The study protocol was examined and approved by the Clinical Research Ethics Committee of Beijing Anding Hospital (Registration Number: 2020-106) and complies with the Code of Ethics of the World Medical Association (Declaration of Helsinki). Informed written consent was obtained from all participants or their legal guardians after a complete and extensive description. Inclusion criteria and exclusion criteria were as follows:

Inclusion criteria for participants with MDD: (1) 18–65 years old (including 18- and 65-years-old), regardless of gender. (2) Meeting the Diagnostic and Statistical Manual of Mental Disorders, 5th edition (DSM-5) diagnostic criteria for MDD, and confirmed by the Mini International Neuropsychiatric Interview (M.I.N.I.) 7.0.2, without psychotic symptoms. (3) A Hamilton Depression Rating Scale-17 (HDRS-17) score of ≥17. (4) No modified electroconvulsive therapy (MECT) within 30 days prior to enrollment. (5) Elementary school education or above, and the ability to understand the scale. (6) Understanding of the research content and provision of written informed consent.

Inclusion criteria for healthy controls (HCs): (1) 18–65 years old (including 18- and 65-years-old), regardless of gender. (2) No previous or current confirmed diagnosis of mental disorder based on M.I.N.I. 7.0.2 screening. (3) Elementary school education or above, and the ability to understand the scale. (4) Understanding of the research content and provision of written informed consent.

Exclusion criteria for participants: (1) A prior diagnosis of bipolar disorder, schizophrenia, schizoaffective disorder, or mental disorder associated with other illnesses. (2) Previous or current patients of organic brain damage such as epilepsy or other disorders in which random brain discharges are present, or serious physical illnesses that make enrollment in this study inappropriate. (3) Having a history of alcohol or psychoactive substance abuse or dependence within 1 year.

### 2.2 EEG recording

A Neuracle NeuSen EEG/event-related potential (ERP) Monitor (Neuracle Technologies, Inc., Changzhou, China) connected to a 19-channel EEG cap (Tenocom Medical Technologies, Co., LTD, Qingdao, China) was used to record raw EEG signals. Data were obtained from 19 Ag/AgCl electrode channels using the advanced Neuracle system, which operated at a sampling rate of 1,000 Hz. The 19-channel EEG raw signals included Fp1, Fp2, F3, F4, F7, F8, Fz, T3, T4, T5, T6, C3, C4, Cz, P3, P4, Pz, O1, and O2. While referencing the Cz electrode, we ensured that impedance was maintained below 50 kΩ. The EEG recording was taken in a quiet and confined room, and participants were asked to sit in a comfortable chair, remaining relaxed and awake. During the process, participants were first asked to face a monitor placed 100 cm away with a black background and stare at a white fixation cross located at the central line of sight for 10 min. Then, in the next 10 min, they were required to close their eyes. EEG was recorded during these two periods (eyes-open, EO; eyes-closed, EC). Participants were asked to remain quiet and relaxed, minimizing head and limb movements. Any movement, dozing, or talking/whispering during the process was immediately corrected and accurately recorded.

### 2.3 Data preprocessing

#### 2.3.1 Preprocessing

**Figure 1:**
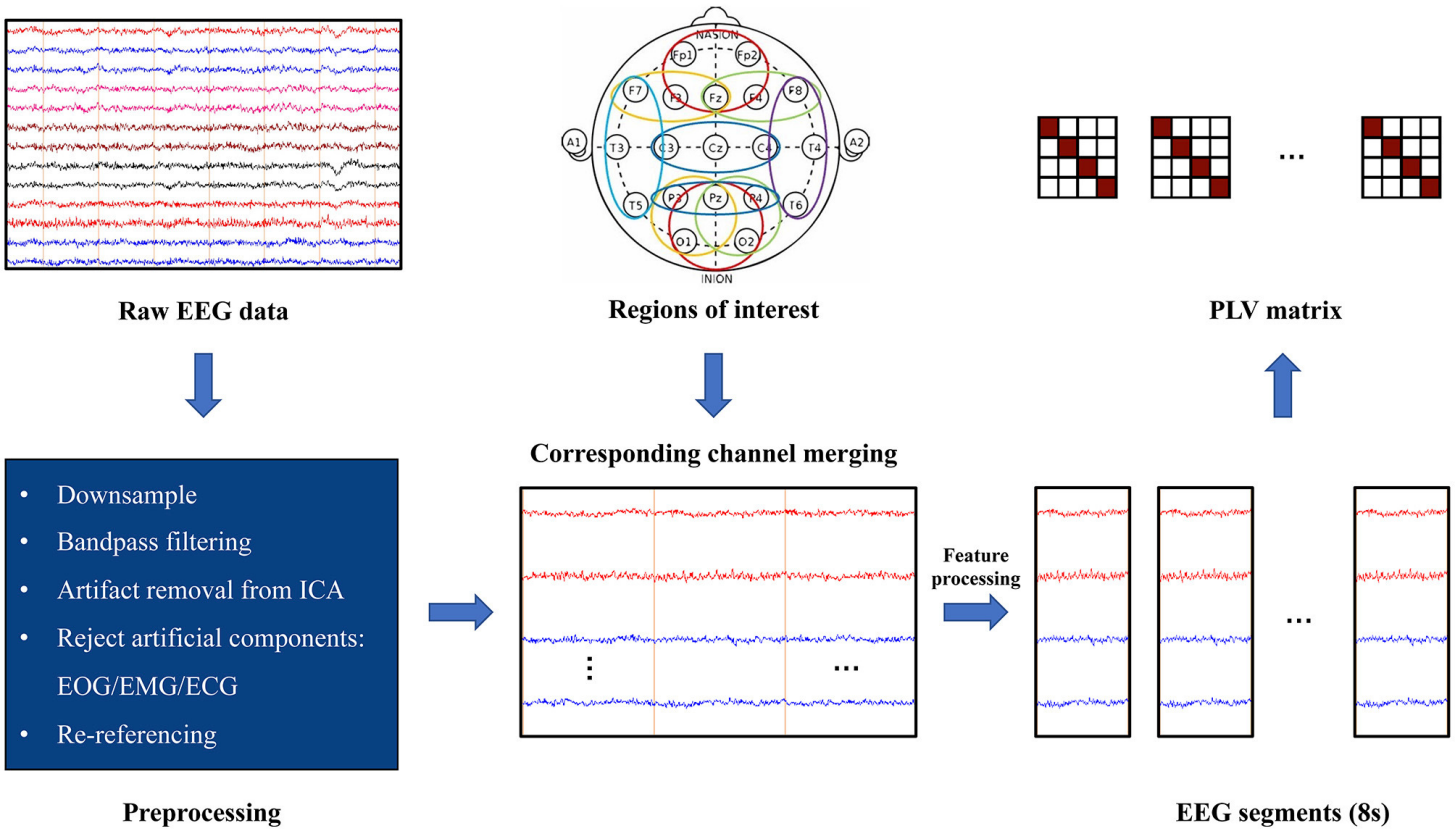
Data preprocessing and PLV calculation.

The EEGLAB toolbox in MATLAB R2013a was used to preprocess the original EEG data. The steps included the following: Downsampled to 256 Hz and bandpass filtered into 1–40 Hz using a finite impulse response (FIR) filter with a hamming window. The period when the amplitudes were larger than 150 μV was removed. Independent component analysis (ICA) was conducted on the remaining data to extract artificial components, including electrooculogram, electromyography, and electrocardiogram. After calculating the ICA components, the MNE-ICA label (Pion-Tonachini et al., 2019) was used to identify and remove artificial components. The data were then averaged and re-referenced to obtain the preprocessed data (Figure 1).

#### 2.3.2 Feature processing

Based on the findings of previous studies, the following five bands were selected: theta (4–8 Hz), alpha (8–12 Hz), beta1 (12–16 Hz), beta2 (16–24 Hz), and beta3 (24–40 Hz). We utilized existing electrodes to divide brain regions, including prefrontal cortex, PFC (Fp1, Fp2, and Fz); right midline frontal cortex, RMFC (Fz, F4, and F8); left midline frontal cortex, LMFC (Fz, F3, and F7); central cortex, CC (C3, C4, and Cz); parietal cortex, PP (P3, P4, and Pz); left temporal cortex, LT (F7, T3, and T5); right temporal cortex, RT (F8, T4, and T6); midline occipital cortex, MOC (O1, Pz, and O2); right midline occipital cortex, RMOC (P4, O2, and Pz); and left midline occipital cortex, LMOC (P3, O1, and Pz).

Furthermore, feature processing was performed on the data after initial preprocessing (Figure 1): The merged signals of each region were filtered into 5 frequency bands by an FIR filter, and then the signal in each band was transformed to a complex signal using the Hilbert transform. The mean value of the complex signals of the assigned channels was calculated on each brain region as the signal of that region. They were sliced into pieces (stride: 8s, overlap: 7s). The average mode length (amplitude) of each 8s segment was evaluated: the mode lengths of the signals in three frequency bands were taken and added together. Then, the amplitudes of the signals in all the brain regions were added, and the average over the length of time was calculated. Anomalous 8s slices of amplitude were removed.

### 2.4 FC calculation (PLV)

PLV, as one of the coupling methods for constructing an FC matrix, mainly assesses the significance of the phase covariance between two signals. It depends on the instantaneous phase of signals (Lachaux et al., 1999). First, it requires filtering of the data in the frequency of interest, followed by extraction of the instantaneous phase using the Hilbert transformation. The calculation method was stated as follows:


(1) $$\begin{array}{l} PLV=|\frac{1}{N}\overset{N}{\underset{n=1}{\sum }}\exp\left(i\left[\phi_{1}\left(n\right)-\phi_{2}\left(n\right)\right]\right)| \end{array}$$

**Figure 2:**
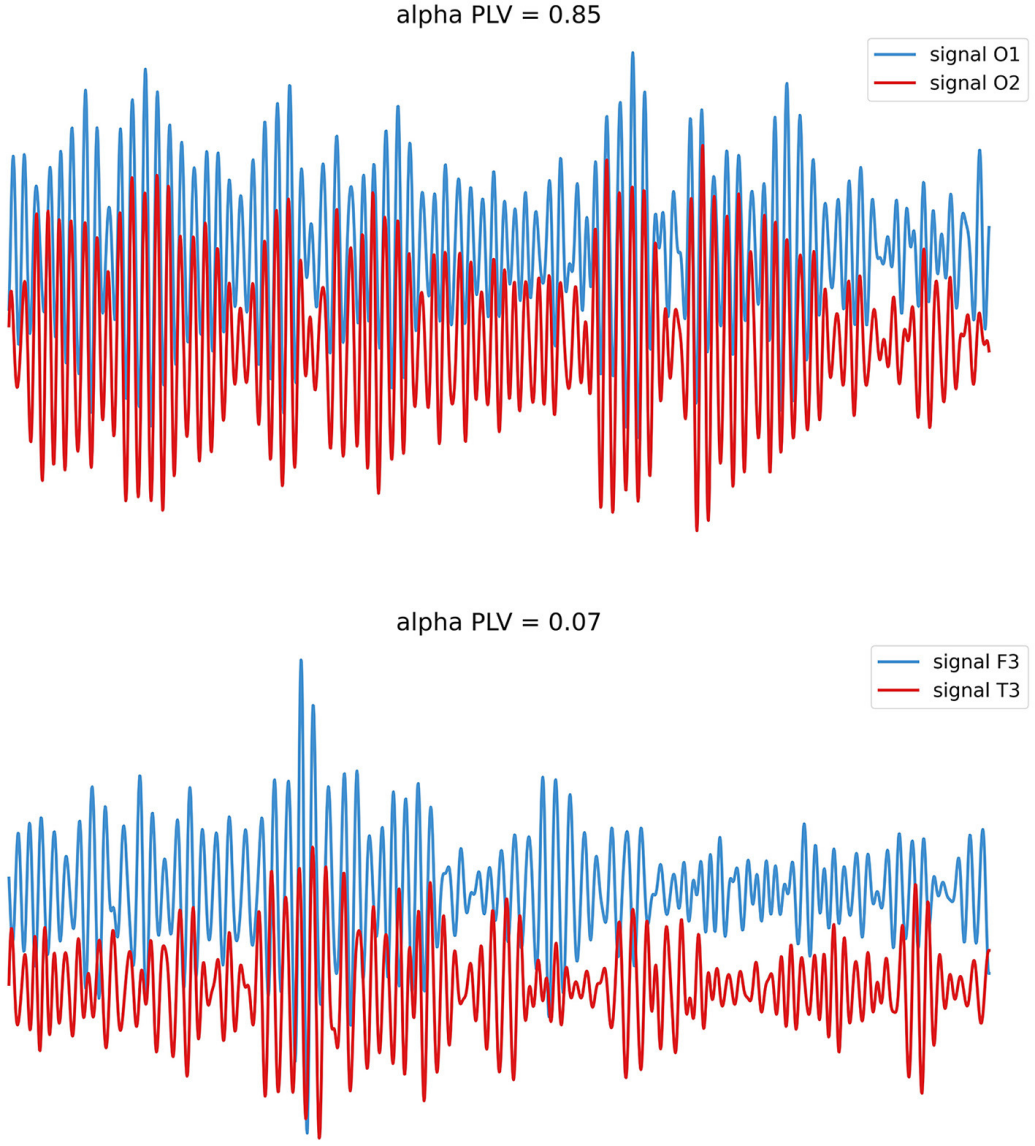
EEG with different levels of PLV. Neural oscillations may perform phase synchronization (above; stable phase relationships) or show rare/no phase synchronization (below; variable phase relationships).

In this formula, *N* represents the length of the time series, and ϕ_1_ (*n*) and ϕ_2_ (*n*) separately refer to the instantaneous phase of two signals at time point *n* (Tan et al., 2022). The process of measure is set up on the assumption of permanent differences between regions, and the PLVs range from 0 to 1 represents the connection strength in a weighted network analysis (Fell and Axmacher, 2011). Figure 2 shows EEG signals with different values of PLV, representing different levels of connection and synchronization in neuronal activities spatiotemporally.

### 2.5 Symptoms evaluation

The severity of symptoms was assessed with HDRS-17 and the Hamilton Rating Scale for Anxiety (HAMA). The Young Mania Rating Scale was completed by participants at the time of screening, which aimed to exclude subjects with manic or hypomanic episodes.

### 2.6 Statistical analysis

All data were analyzed using SPSS 26.0 software (IBM, Armonk, NY, USA). Continuous data were tested for normality. Normally distributed continuous data are expressed as mean ± standard deviation (SD), non-normally-distributed continuous data are expressed as median (interquartile range), and categorical data are expressed as *n* (%). Group differences in continuous data were calculated by conducting a *t*-test or Mann–Whitney *U*-test depending on their normality, while for categorical variables, the differences were examined by conducting χ^2^ analyses. The relationship between the PLV and HDRS scores was assessed using Spearman's correlation coefficient because of the non-normal distribution of the HDRS data. The significance level in this study was set to 0.05; however, there were multiple comparisons between brain regions, so the Bonferroni method was used to correct the significance of the *p*-value. The final significance was set to *p* < 0.001 when comparing the connectivity index between the two groups.

## 3 Results

### 3.1 Demographic information

The present study was conducted in parallel with Liu et al.'s research, and the socio-demographic information is consistent with that presented in their study (Liu et al., 2024). A total of 169 participants were enrolled in this study, of which 86 were recruited in the MDD group and 83 in the HC group. There was no significant difference in age, gender, current marital status, and education years (all *p* > 0.05). This indicated that the demographic characteristics of the enrolled subjects in the two groups were matched and comparable.

### 3.2 PLV

We calculated the connections between the 10 brain regions sequentially and compared the MDD group with the HC group.

#### 3.2.1 EO

**Figure 3:**
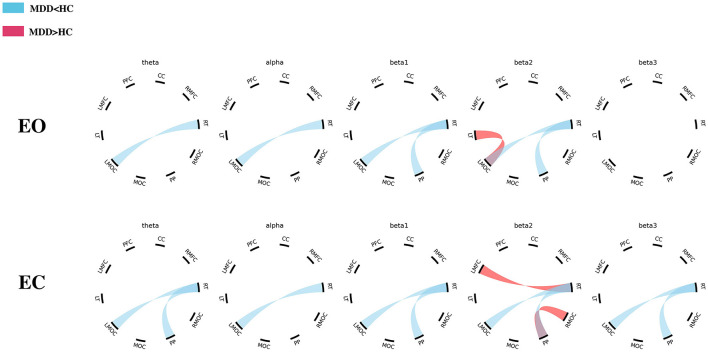
The differences in EEG PLV between the MDD and HC groups (EO/EC). MDD, major depressive disorder; HC, healthy control; PFC, prefrontal cortex; CC, central cortex; RMFC, right midline frontal cortex; RT, right temporal cortex; RMOC, right midline occipital cortex; PP, posterior parietal cortex; MOC, midline occipital cortex; LMOC, left midline occipital cortex; LT, left temporal cortex; LMFC, left midline frontal cortex.

During the EO phase, there were lower PLVs in the connections of right temporal-left midline occipital cortex (RT-LMOC; theta, alpha, beta1, and beta2) and posterior parietal-right temporal cortex (PP-RT; beta1 and beta2) in the MDD group than those in the HC group. Moreover, PLVs were higher in the beta2 band in the MDD group than those in the HC group when the connections of LT-LMOC were observed (Table 1, Figure 3).

**Table 1:** The differences in EEG PLV between the MDD and HC groups (EO).

		**MDD**	**HC**	***Z/t***	***p***
		**(*n*=86)**	**(*n*=83)**		
LT-LMOC	beta2	0.188 (0.122, 0.259)	0.133 (0.106, 0.185)	−3.497^***^	0.000
PP-RT	beta1	0.248 (0.156, 0.364)	0.342 (0.241, 0.459)	−3.509^***^	0.000
	beta2	0.166 (0.122, 0.274)	0.258 (0.172, 0.397)	−3.739^***^	0.000
RT-LMOC	theta	0.245 (0.171, 0.343)	0.323 (0.246, 0.448)	−3.755^***^	0.000
	alpha	0.338 ± 0.136	0.414 ± 0.138	−3.604^***^	0.000
	beta1	0.333 ± 0.135	0.418 ± 0.132	−4.114^***^	0.000
	beta2	0.235 (0.152, 0.338)	0.335 (0.247, 0.425)	−4.050^***^	0.000

Due to data and space limitations, this table presents only the brain region connections where differences were observed.

MDD, major depressive disorder; HC, healthy control; LT, left temporal cortex; RT, right temporal cortex; PP, posterior parietal cortex; LMOC, left midline occipital cortex.

^***^p < 0.001.

#### 3.2.2 EC

During the EC period, there were lower theta and beta (beta1, beta2, and beta3) PLVs in the connections of PP-RT in the MDD group than those in the HC group. There were also lower theta, alpha, and beta (beta1, beta2, and beta3) PLVs in the connections of RT-LMOC in the MDD group than those in the HC group. Additionally, in the left midline frontal cortex-right temporal cortex (LMFC-RT) and posterior parietal cortex-right temporal cortex (PP-RMOC), higher PLVs were found in the frequency of beta2 (Table 2, Figure 3).

**Table 2:** The differences in EEG PLVs between the MDD and HC groups (EC).

		**MDD**	**HC**	***Z/t***	***p***
		**(*****n***=**86)**	**(*****n***=**83)**		
LMFC-RT	beta2	0.251 (0.150, 0.330)	0.162 (0.107, 0.267)	−3.774^***^	0.000
PP-RMOC	beta2	0.742 (0.677, 0.782)	0.695 (0.650, 0.734)	−3.862^***^	0.000
PP-RT	theta	0.179 (0.145, 0.230)	0.237 (0.183, 0.345)	−4.057^***^	0.000
	beta1	0.222 (0.163, 0.283)	0.275 (0.213, 0.407)	−3.921^***^	0.000
	beta2	0.154 (0.117, 0.212)	0.236 (0.170, 0.298)	−5.371^***^	0.000
	beta3	0.141 (0.096, 0.208)	0.216 (0.151, 0.289)	−4.494^***^	0.000
RT-LMOC	theta	0.240 ± 0.073	0.302 ± 0.102	−4.482^***^	0.000
	alpha	0.316 (0.271, 0.382)	0.403 (0.316, 0.477)	−4.006^***^	0.000
	beta1	0.342 ± 0.102	0.426 ± 0.121	−4.888^***^	0.000
	beta2	0.289 ± 0.100	0.379 ± 0.119	−5.303^***^	0.000
	beta3	0.281 ± 0.125	0.376 ± 0.156	−4.401^***^	0.000

Due to data and space limitations, this table presents only the brain region connections where differences were observed.

MDD, major depressive disorder; HC, healthy control; LMFC, left midline frontal cortex; RT, right temporal cortex; PP, posterior parietal cortex; RMOC, right midline occipital cortex; LMOC, left midline occipital cortex.

^***^p < 0.001.

### 3.3 Correlation analysis

We calculated and statistically correlated the above differential connections' PLVs with the HDRS scores of participants with MDD. The purpose was to verify the relationship between FC and participants' depression levels. After calculation, there were no significant correlations between PLVs and HDRS scores when the connections were examined with significantly different PLVs (all *p* > 0.05).

## 4 Discussion

In our study, the brain of each participant was categorized into 10 regions of interest according to the orientation of the electrodes. These brain regions cover all parts of the cerebral cortex. We followed this partitioning method to further analyze and compare functional connections between patients with MDD and HCs. The results showed that during the EO period, PLVs of the MDD group were lower than those of the HC group in PP-RT (beta2) and RT-LMOC (theta, alpha, beta1, and beta2), while in LT-LMOC (beta2), the PLVs were higher in the MDD group than in the HC group. During the EC period, PLVs of PP-RT (theta, beta1, beta2, and beta3) and RT-LMOC (theta, alpha, beta1, beta2, and beta3) were found to be lower in the MDD group than in the HC group. However, in the connections of LMFC-RT and PP-RMOC, the PLVs of patients with MDD were demonstrated to be higher than that of HCs in the beta2 band.

We measured and analyzed FC by calculating PLVs in different frequency bands. As mentioned in the introduction, synaptic signals of different frequencies are often linked with top-down inhibitory processes. In EEG studies, these frequencies usually have their own significance in individuals with MDD. Specifically for this research, we analyzed theta, alpha, and beta bands, among which the change in theta and alpha were thought to be the results of white matter dysfunction (Nunez et al., 2015). More specifically, for the alpha band, such violation might disrupt the ability of patients with MDD in terms of attention and executive functions (Miljevic et al., 2023), while for the theta band, the ability of cognitive control could be weakened (Cavanagh and Frank, 2014). Moreover, intrinsically motivated decision-making was associated with the theta and beta bands (Nakao et al., 2012). The following is a discussion and further analysis of our results for the different frequency bands.

### 4.1 Theta and alpha FC

In past studies, more attention has been given to theta and alpha frequency bands. Most of the studies indicate that resting-state alpha FC is higher in people with depression; more specifically, there are more instances of higher alpha FC in frontal regions and lower FC in the parietal-occipital area. However, for theta bands, there is no consistent conclusion because of the different methods of FC analysis (Leuchter et al., 2012; Ahn et al., 2017; Fingelkurts and Fingelkurts, 2017; Iseger et al., 2017; McVoy et al., 2019; Hasanzadeh et al., 2020; Dell'Acqua et al., 2021; Miljevic et al., 2023). In our study, during EC, FC associated with RT (PP-RT and RT-LMOC) was lower in participants with MDD in the theta band, and alpha FC was also lower in RT-LMOC. For EO, PLVs of RT-LMOC were lower in the MDD group in the theta and alpha bands, which was the same as EC. For alpha, different from previous studies, our findings focused more on the connections of the temporal cortex and occipital area (RT-LMOC). The PLVs of participants with MDD were lower in both EC and EO. It has been pointed out that the temporal lobe plays a crucial role in MDD. Fan et al.'s resting-state functional magnetic resonance imaging (fMRI) study found that aberrant right superior temporal gyrus (STG) activity might be a potential marker of suicide attempts among patients with MDD (Fan et al., 2013). Blackhart et al.'s study indicated that less right parieto-temporal activity is correlated with more severe symptoms of depression (Blackhart et al., 2006). These findings confirm the rationality and validity of our results to a certain extent; abnormal activity in the right temporal lobe may weaken its FC with other brain regions. For the theta band, our results showed lower PLVs in the right temporal-parietal/left occipital region, which has been linked with the decrease of cognitive control efficacy of related top-down processes (Hwang et al., 2015), and lower theta FC was found to be associated with depressive symptoms (McVoy et al., 2019). However, as mentioned before, there are various findings related to theta FC. In the study conducted by Leuchter et al., the resting-state EEG of 121 unmedicated participants with MDD and 37 HCs was included in the analysis. The results showed that the coherences of the participants with MDD were higher than those of HCs between the frontopolar and temporal/parietooccipital regions (Leuchter et al., 2012). Another research that applied phase transfer entropy as the measure of phase-based effective connectivity also indicated a higher node degree and strength in the directed differential connectivity graph (dDCG) of participants with MDD than HCs (Hasanzadeh et al., 2020). Nevertheless, the results of an EEG study among adolescents revealed that the average coherence of the MDD cohort in the theta band was lower than that of HCs; researchers attributed this to the delayed maturation of the default mode network in youth with MDD (McVoy et al., 2019). However, other studies have also found lower theta FC in individuals with MDD. According to the results of Knott et al., patients with MDD exhibited smaller theta coherence values than HCs (Knott et al., 2001). In another pilot study aimed at comparing resting-state EEG coherence in somatic symptom disorder and MDD, researchers found that theta coherence between T5-P3 electrodes (the left temporoparietal junction, which has been linked with cognitive-attentional processing and social interaction) was lower in the MDD group than in the HC group (Ahn et al., 2017). In other words, it is difficult to form a consistent conclusion about the theta frequency band. Future studies with the usage of higher-quality methodological steps are needed to further investigate the differences between individuals with MDD and HCs in theta FC.

### 4.2 Beta FC

For the beta frequency, there also never seemed to be a consensus. According to a graph theory analysis conducted by Hasanzadeh et al., after calculating the density, degree, and strength of directed dDCG networks in all frequency bands for normal and MDD groups, the researchers found higher density and strength in beta1 (13–16 Hz). This indicated that there were more links in MDD networks and that their weights were significantly higher than those of the corresponding links in the normal group (Hasanzadeh et al., 2020). Leuchter et al. found higher beta coherence in participants with MDD, primarily in connections within and between electrodes overlying the dorsolateral prefrontal cortical or temporal regions (Leuchter et al., 2012). For the measurement of coherence, another study investigated changes in interhemispheric coherence in different neuropsychiatric disorders. The researchers found that patients with depression showed significantly greater interhemispheric beta coherence in C3-C4 than the control group in both EC and EO conditions (Markovska-Simoska et al., 2018). Different from the results aforementioned, some previous studies stated that the beta FC of participants with depression was lower than that of HCs, such as the study by Knott et al. They compared the coherence measures derived from spectrally analyzed EEGs, and they found that beta coherence was lower in patients with MDD than in HC, regardless of inter-hemispheric or intra-hemispheric differences (Knott et al., 2001). McVoy claimed that beta coherence in adolescents with MDD is significantly lower in the connections of P3-O1 and Fp2-F4; nonetheless, the researcher ascribed the findings to the developmental retardation of the brain (McVoy et al., 2019). In our research, PLVs of beta2 (16–24 Hz) were found to be higher in the region pairs of LT-LMOC (EO), LMFC-RT (EC), and PP-RMOC (EC) in participants with MDD than in HCs, whereas in the connections of PP-RT and RT-LMOC, the FCs were all relatively lower to varying degrees in the beta band. It can be inferred that results presented in the beta band would be diverse depending on their brain area connections. Among beta connections, the frequency of beta2 existed in each connection with significant differences between participants with MDD and HCs. In the study conducted by Huang et al., higher beta2 coherence was observed in participants with MDD than in HCs, including the coherence between the left PFC and right amygdala (F7–T4) and the index between the right PFC and left amygdala (F8–T3; Huang et al., 2023). Looking back at our findings, the PLV of LMFC-RT (EC) was higher in the MDD group than in HCs, which was spatially consistent with the earlier result. However, the previous coherence study was limited to the frontal-limbic circuit, and only a few studies on the beta FC of MDD have focused on specific brain regions; the quality of assessment in these studies also varied. Therefore, considering this and the fuzzy sub-band divisions in the existing studies, further consensus should be reached on methodological steps for obtaining more precise and normatively consistent conclusions.

### 4.3 The connection of RT-LMOC

We found that in the connection of RT-LMOC, the region pair had lower PLV in almost all the frequency bands, regardless of the EO or EC condition. Previous studies have accustomed us to focus on the FC of a particular electrode pair in a specific frequency band, although witnessing such consistent FC changes was fairly rare. The low functional connection of the right temporal with the left middle occipital region might be one of the critical features of brain dysfunction in patients with MDD. It is known that the temporal lobe contributes to the abilities of language, memory, senses, and emotion, determining how we experience and process certain emotions; the occipital lobe, located at the rearmost position, is mainly responsible for visual processing. More specifically, this part processes visual signals and works collaboratively with other brain regions. It plays a vital role in language and reading, memory storage, and the recognition of familiar objects, such as places or faces. In a meta-analysis study, researchers applied activation likelihood estimation, a method with ideal spatial sensitivity that can implement voxel-wise statistical comparison of numerous studies, and found that the right STG was the largest among nine significant clusters when analyzing the activation foci associated with happiness. For the same analysis related to greater happiness than sadness, the largest cluster was also located in the right STG, while in the analysis related to greater sadness than happiness, the largest cluster was found in the right middle temporal gyrus.

These results highlighted the significance of the right temporal gyrus in the generation of happy or sad emotions; the lower PLVs of RT-LMOC, which might be a reflection of the process, further established the strong association between depressive mood and the right temporal region (Vytal and Hamann, 2010). An earlier study demonstrated through tractography a direct connection from the extrastriate occipital cortex to the anterior temporal region, as well as indirect connections of the occipital-temporal projection system (Catani et al., 2003). The deficit in these connections might affect the ability to learn novel, non-verbalizable visual stimuli, which is speculated to be the direct way to prime medial temporal structures and facilitate the consolidation of visual memories. This indicated that the lower FC of RT-LMOC might be linked with cognitive dysfunctions in vision-related domains. According to an fMRI study, the middle longitudinal fascicle (MdLF), which is known as the fiber tract that links different parts of temporal lobes, has been validated due to broader connections, including the temporo-occipital region (Makris et al., 2017). Such connections might be related to language, attention, and visual and auditory processing functions; thus, the disruption of the MdLF has been linked with several neuropsychiatric disorders, which might further lead to aphasia, behavioral variants, and attention-deficit disorders. This finding verified the existence of a temporo-occipital connection and its relationship with psychiatric disorders. In our study, the connection of RT-LMOC was found to be weaker in participants with MDD, and the results are consistent with previous findings to a certain extent. However, previous findings were mostly based on the results of cognitive assessments or paradigms in ERP studies, while our study was conducted under resting-state conditions. Thus, future studies must design and utilize some paradigms that can reveal different domains of cognitive functions to further discover the connections of the temporo-occipital region.

### 4.4 PLV and MDD severity: no correlation

We conducted a correlation analysis between the PLVs of different connections in the two groups and their HDRS-17 scores. The results did not show any significant correlation, which means that the value of PLV could not be determined or that it was immediately affected by the severity of depression. The possible reason might be that FC measured by PLV is more likely to be a persistent trait marker of MDD, indicating that spatiotemporal synchronization between different brain regions could be long-lasting or at least not easily affected and altered soon. As mentioned before, FC is influenced by myelinated cortico-cortical axons of the white matter, and myelin controls the speed of the axon and the synchrony of impulse transportation. This suggests that although FC symbolizes instantaneous phase connection, the changes in PLV are based on physical structure alteration; such alteration might not occur as quickly as MDD becomes more severe or attains remission. Finally, during the period of inclusion, we set the HDRS of participants with MDD as no < 17, which means that the patients involved in this study had moderate to severe depression; therefore, the range of scores was relatively narrow when compared to other studies. In future studies, larger-sampled follow-up research is essential to further verify such assumptions. The effect of medical treatment should also be taken into consideration.

### 4.5 Highlights and limitations

This study is innovative and highlights FC in MDD. In our research, we applied a new classification method of brain regions to demarcate 10 regions of interest and offered a comprehensive and balanced view of the changes in function across different areas of the brain. In terms of FC, different from the connectivity values between electrode pairs, this method of taking the mean value of the assigned channels reflects, to some extent, FC between corresponding brain compartments. In addition, resting-state EEG was observed during the EO and EC phases, which would allow for a broader range of observations because the brainwave patterns of the two states themselves differ in certain frequency bands. This might be due to the different visual sensory information and subjective/objective state characteristics of these two conditions, which might be related to an exteroceptive network and an interoceptive network (Tan et al., 2013; Xu et al., 2014).

Some limitations of this study should be noted. First, although the basic information of the two groups of participants was carefully matched and it was verified that there were no statistically significant differences in the mentioned data, the results of the study are still limited due to the lack of further analysis of some other confounding factors, such as patients' gender, age, and education level. Thus, whether FC was related to these factors could not be determined. Second, we did not further consider the effect of comorbidities, such as anxiety disorders, and their presence might also confound the EEG signals. Finally, as mentioned before, this cross-sectional study lacks follow-up data; whether altered FC is a state marker that can recover after treatment or a persistent trait marker of MDD remains unclear. Large-sampled and long-term controlled studies are still required to further discover the characteristic changes in the EEG of patients with MDD and their relation to the pathogenesis of depression.

## 5 Conclusions

Compared with healthy individuals, patients with MDD tend to have lower PLV in the connection of the right temporal and left occipital lobes in most cases. However, an increase in PLV can be found in the connection of the left temporal with the left occipital lobe in patients with MDD (EO). During EC, an increase can also be found in the circuits of the frontal-temporal and parietal-occipital regions. The trends in FC observed in this study were not correlated with the level of depression.

## Data availability statement

The raw data supporting the conclusions of this article will be made available by the authors, without undue reservation.

## Ethics statement

The studies involving humans were approved by Clinical Research Ethics Committee of Beijing Anding Hospital. The studies were conducted in accordance with the local legislation and institutional requirements. The participants provided their written informed consent to participate in this study.

## Author contributions

YW: Data curation, Formal analysis, Investigation, Methodology, Software, Supervision, Validation, Writing – original draft, Writing – review & editing. YCh: Data curation, Formal analysis, Investigation, Methodology, Software, Supervision, Validation, Writing – original draft, Writing – review & editing. YCu: Methodology, Project administration, Software, Supervision, Validation, Visualization, Writing – review & editing. TZ: Methodology, Software, Supervision, Validation, Visualization, Writing – review & editing. BW: Data curation, Project administration, Resources, Software, Validation, Visualization, Writing – review & editing. YZ: Formal analysis, Investigation, Software, Supervision, Validation, Writing – review & editing. YR: Methodology, Project administration, Resources, Supervision, Validation, Visualization, Writing – review & editing. SS: Methodology, Project administration, Resources, Supervision, Validation, Visualization, Writing – review & editing. YY: Project administration, Resources, Writing – review & editing. XZ: Conceptualization, Data curation, Formal analysis, Funding acquisition, Investigation, Methodology, Project administration, Resources, Supervision, Validation, Visualization, Writing – review & editing. LZ: Conceptualization, Investigation, Methodology, Project administration, Resources, Supervision, Validation, Visualization, Writing – review & editing. GW: Conceptualization, Investigation, Methodology, Project administration, Resources, Supervision, Validation, Visualization, Writing – review & editing.
